# Addressing identification bias in the design and analysis of cluster-randomized pragmatic trials: a case study

**DOI:** 10.1186/s13063-020-4148-z

**Published:** 2020-03-23

**Authors:** Jennifer F. Bobb, Hongxiang Qiu, Abigail G. Matthews, Jennifer McCormack, Katharine A. Bradley

**Affiliations:** 1 Biostatistics Unit, Kaiser Permanente Washington Health Research Institute, 1730 Minor Ave, Seattle, WA 98101 USA; 2 Department of Biostatistics, University of Washington, 1705 NE Pacific St, Seattle, WA 98195 USA; 3 The Emmes Company, 401 N Washington St # 700, Rockville, MD 20850 USA; 4 Department of Health Services, University of Washington, 1959 NE Pacific St, Seattle, WA 98195 USA; 5 Department of Medicine, University of Washington, 1959 NE Pacific St, Seattle, WA 98195 USA

**Keywords:** Electronic health records, Recruitment bias, Identification bias, Cluster-randomized trials, Opioid use disorder, Implementation trial, Open-cohort trial

## Abstract

**Background:**

Pragmatic trials provide the opportunity to study the effectiveness of health interventions to improve care in real-world settings. However, use of open-cohort designs with patients becoming eligible after randomization and reliance on electronic health records (EHRs) to identify participants may lead to a form of selection bias referred to as *identification bias*. This bias can occur when individuals identified as a result of the treatment group assignment are included in analyses.

**Methods:**

To demonstrate the importance of identification bias and how it can be addressed, we consider a motivating case study, the PRimary care Opioid Use Disorders treatment (PROUD) Trial. PROUD is an ongoing pragmatic, cluster-randomized implementation trial in six health systems to evaluate a program for increasing medication treatment of opioid use disorders (OUDs). A main study objective is to evaluate whether the PROUD intervention decreases acute care utilization among patients with OUD (effectiveness aim). Identification bias is a particular concern, because OUD is underdiagnosed in the EHR at baseline, and because the intervention is expected to increase OUD diagnosis among current patients and attract new patients with OUD to the intervention site. We propose a framework for addressing this source of bias in the statistical design and analysis.

**Results:**

The statistical design sought to balance the competing goals of fully capturing intervention effects and mitigating identification bias, while maximizing power. For the primary analysis of the effectiveness aim, identification bias was avoided by defining the study sample using pre-randomization data (pre-trial modeling demonstrated that the optimal approach was to use individuals with a prior OUD diagnosis). To expand generalizability of study findings, secondary analyses were planned that also included patients newly diagnosed post-randomization, with analytic methods to account for identification bias.

**Conclusion:**

As more studies seek to leverage existing data sources, such as EHRs, to make clinical trials more affordable and generalizable and to apply novel open-cohort study designs, the potential for identification bias is likely to become increasingly common. This case study highlights how this bias can be addressed in the statistical study design and analysis.

**Trial registration:**

ClinicalTrials.gov, NCT03407638. Registered on 23 January 2018.

## Background

Pragmatic clinical trials provide the opportunity to study the effectiveness of interventions in real-world medical settings [1, 2]. Unlike traditional trials in which a highly selected patient population is recruited to participate [3], pragmatic trials can be conducted in entire clinic populations or health systems with study eligibility criteria and outcomes defined using routinely collected information, such as electronic health record (EHR) data [4, 5]. The benefits of pragmatic trials are increasingly being recognized: large sample sizes (and relatively low cost per participant), representativeness of study populations that include subgroups often excluded from clinical research (e.g. youth, pregnant women), and direct relevance of study findings to inform clinical practice [6, 7].

Yet pragmatic trials also introduce several methodological challenges [8–13]. One major challenge in pragmatic cluster-randomized trials occurs when participants are identified after randomization, as in open-cohort or continuous recruitment designs [14–16]. These types of designs can have important advantages in some settings, including efficiency of using data from all individuals exposed to the intervention, and the ability to examine short-term exposures [15, 16]. At the same time, inclusion of participants identified post-randomization in analyses has the potential for selection bias due to the intervention affecting which participants are identified as being eligible [17, 18]. This type of selection bias has been referred to as *identification bias* [18–20] and results from the fact that individuals who are identified in intervention clusters post-randomization are often different from those who are identified in usual care clusters.

This potential for bias is well-recognized in trial settings in which individuals are formally recruited to participate (i.e. recruitment bias [18]). However, its role in pragmatic trials in which there is no patient contact for study recruitment or data collection purposes has been less well appreciated and is often unaddressed, posing a threat to the validity of these trials. Because such studies must identify eligible patients solely using routinely collected data (e.g. EHR data), several of the recommended approaches for addressing recruitment bias, such as having masked recruiters contact potential study participants to ask them about their eligibility [18], are not possible. Consequently, a framework for addressing identification bias in the statistical study design and analysis is needed for these types of pragmatic trials.

In this paper, we illustrate how identification bias poses a threat to valid inferences and propose methods for mitigating this source of bias in the statistical design of the PRimary care Opioid Use Disorders treatment (PROUD) Trial, an ongoing cluster-randomized, open-cohort, pragmatic implementation trial [21] to evaluate a program for increasing medication treatment for patients with opioid use disorders (OUDs) in six health systems. We highlight the tradeoffs of minimizing bias in estimating intervention effects while maximizing the generalizability of study findings and statistical power when the target study population of interest, in this case patients with OUD, is underrecognized. Although developed in the context of the PROUD Trial, the proposed methodology for addressing identification bias can be applied in any pragmatic trial in which the intervention is expected to affect identification of the population under study.

## Methods

### Description of the PROUD Trial

#### Background

Addiction to opioids has reached epidemic proportions in the United States, resulting in increases in drug overdose and death [22, 23]. Although several medications are effective for treating OUD, including methadone, buprenorphine, and injectable naltrexone (the latter two of which can be prescribed in primary care) [24, 25], most people with OUD do not receive treatment [26]. New approaches are being developed to increase access to and retention in evidence-based treatment, especially in primary care [27]. One promising approach is to have a full-time nurse care manager support primary care providers in treating OUDs [28, 29]. A key element of this model is that it attracts new patients into the primary care site who are seeking treatment for OUD [28, 29]. Because many healthcare settings have limited (or sometimes no) access to evidence-based OUD treatment options, patients with OUD may seek out any newly available sources of treatment. Nurse care managers who support treatment for OUD may also increase identification of OUD in patients already receiving primary care at the site (e.g. by destigmatizing OUD treatment).

#### PROUD Study design

PROUD is a pragmatic, cluster-randomized implementation trial to evaluate a program of office-based addiction treatment of OUD in primary care settings. The study funds the health system to hire a nurse care manager (“the nurse”) who coordinates care with the site’s primary care team to provide evidence-based OUD medication treatment [30, 31]. The trial is set within 12 primary care sites in six healthcare systems. Randomization is stratified on the health system, with one site randomized to receive the nurse care manager program (“PROUD intervention”) and the other to receive no intervention: primary care continues as usual (“control arm”). All data for the trial, including data used to identify the study population for inclusion in trial analyses and assess outcomes, come from the EHR and other automated data sources [45] (e.g. administrative datasets); there is no primary data collection for main outcomes and no contact with patients for recruitment or data collection.

#### PROUD effectiveness objective

The trial aims to evaluate whether the PROUD intervention successfully (1) increases evidence-based medication treatment for OUD (implementation evaluation) and (2) improves health outcomes among patients with OUD (effectiveness evaluation). To illustrate methods for addressing identification bias, the current case study focuses on this latter effectiveness aim, where the optimal approach to addressing identification bias was not clear. The effectiveness outcome is a patient-level measure of the number of days of acute care utilization (including hospitalizations, emergency department visits, and urgent care), an adverse health outcome among patients with OUD.

#### PROUD study sample

The study includes an open-cohort sample of patients with a primary care visit to one of the randomized sites during the period from three years before randomization (“baseline period”) until two years after randomization (“follow-up period”). However, as we will discuss below, the analytic sample for our effectiveness objective was restricted to a subset of this cohort.

#### Statistical analysis plan for the PROUD effectiveness objective

To model the patient-level number of days of acute care utilization over the follow-up period, the planned analysis is to apply a Poisson mixed-effect model with a random intercept for site that adjusts for pre-specified covariates known (or hypothesized) to be associated with acute care utilization, such as the baseline value of the outcome [30, 31].

### Design and analytic challenges related to identification bias

**Fig. 1:**
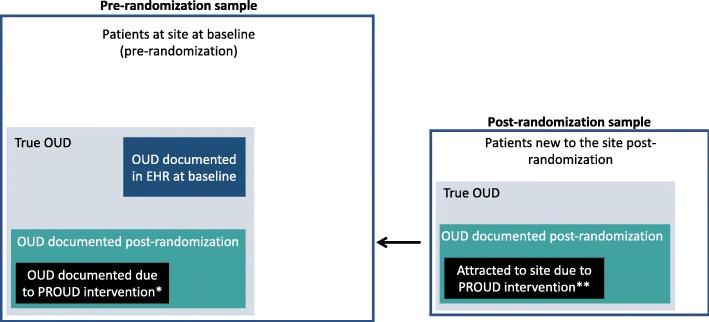
Analytic samples available for inclusion in analyses of PROUD intervention effects before and after randomization. Boxes not drawn to scale. * Increase in documentation of an OUD diagnosis may be due to increased skill in diagnosing and treating OUD or increased patient disclosure due to reduced stigma. ** Includes patients who are attracted to the intervention site because they are seeking OUD treatment (e.g. due to limited access to treatment elsewhere or lower barriers to receiving care in the PROUD intervention site)

Because there is no patient contact for research purposes, eligibility criteria for identifying the population of patients to be included in trial analyses must be defined solely using automated data sources as discussed above. However, identifying the target population of interest (patients with OUD) is difficult, because OUD is underrecognized within general primary care populations and, in particular, is frequently not documented in the EHR (see Fig. 1). Based on preliminary studies conducted before the trial (PROUD Phase 1), about 0.5% of the approximately 300,000 primary care patients seen over a three-year period in the 12 PROUD sites had an active OUD diagnosis documented in the EHR, whereas the true prevalence of OUD is thought to be in the range of 1%–4% in general primary care populations (with higher rates in certain subpopulations) [26, 32–35]. Moreover, because OUD is underdiagnosed, patients who have OUD documented in their EHR may not reflect the broader population of patients with OUD. To distinguish between patients with an OUD diagnosis documented in the EHR and patients who have OUD regardless of its documentation in the EHR, we will use the terms “documented OUD” and “true OUD,” respectively. Similarly, we will refer to the corresponding proportions of patients as the “prevalence of documented OUD” and the “true prevalence” of OUD.

Additionally, the PROUD intervention is expected to increase identification of OUD, both by increasing diagnosis of OUD among individuals who are already receiving primary care in the site due to nurse assessments, as well as by attracting individuals with OUD who are seeking OUD treatment into primary care who have not been seen previously at the intervention site (Fig. 1). These latter individuals could be attracted from a different site in the health system or may be new to the health system entirely. As part of the nurse care manager program, individuals being treated for OUD typically receive a documented diagnosis in their EHR. In prior instances where this program was implemented, it was observed that a high proportion of patients being treated by the nurse were new to the site [28, 29] for reasons outlined above.

The under-documentation of OUD in the EHR, together with the likelihood that the intervention increases documentation of OUD and attracts new patients with OUD to the site, makes defining eligibility criteria for inclusion in statistical analyses of PROUD trial outcomes challenging. Restricting analyses to individuals identified pre-randomization avoids the potential for identification bias at the cost of missing benefits to patients (i.e. decreased acute care utilization) that result from the intervention. In particular, such an approach would miss the benefits to new patients who are attracted by the nurse care manager program because of a lack of treatment options elsewhere, or patients who only disclosed their OUD symptoms to clinicians (who then documented this newly disclosed OUD in the EHR) after treatment was available. On the other hand, allowing individuals to be identified for inclusion in the sample post-randomization, as is often done in open-cohort designs, introduces the potential for estimates of intervention effects to be biased. Table 1 uses a hypothetical example to illustrate how including patients identified post-randomization in an analysis could adversely impact a study, leading to identification bias and potentially even an incorrect conclusion (though a thoughtful analytic plan together with adequate covariate information could mitigate the potential for bias).

**Table 1:** Hypothetical example illustrating identification bias^a^ when individuals identified using post-randomization data are included in analyses

Research Question: Does the intervention decrease the number of days of acute care utilization among patients with OUD (patient-level outcome)?	
Assumption: Assume the intervention has no effect on reducing acute care utilization Analytic sample: An open cohort of individuals with an OUD diagnosis documented in the EHR (pre- and/or post-randomization) Patients identified using pre-randomization data: • Suppose the number of patients with documented OUD pre-randomization is 100 in each trial arm (control and intervention) • Assume an average of 9 days of acute care utilization per year at baseline among these patients with OUD in each arm Patients identified using post-randomization data: • Control: 25 patients receive a new documented OUD diagnosis post randomization. These patients have an average of 9 days of acute care per year at baseline • Intervention: 50 patients receive a new documented OUD diagnosis. Of these, 25 are diagnosed as part of the intervention program and the other 25 are diagnosed through other mechanisms as in the control sites • Suppose patients diagnosed via the intervention program are sicker as compared to those diagnosed through other mechanisms, with an average of 12 days of acute care per year (versus 9) at baseline Estimated intervention effect: • Control: among 125 patients with a diagnosis, there is an average of 9 days of acute care per year of follow-up^b^ • Intervention: among 150 patients with a diagnosis, there is an average of 9.5 days of acute care per year of follow-up^b^ [= 9*125/150 + 12*25/150] Summary: We would estimate that the intervention results in greater acute care utilization relative to control, even if there is truly no effect. The bias could go in the other direction if patients diagnosed as part of the intervention program are healthier (rather than sicker) than patients diagnosed through other mechanisms.	

*EHR* electronic health record, *OUD* opioid use disorder

^a^ Identification bias is a form of selection bias that can occur in open-cohort cluster-randomized trials when the randomized intervention group assignment affects who is identified as eligible for a particular analysis

^b^ For simplicity, here we assume no time trend (i.e., that average number of days of acute care per year of follow up is the same as the average per year at baseline)

### Framework to address identification bias in the statistical design

An initial step in the statistical design for a particular study objective is to first determine whether the objective is susceptible to identification bias (Step 0). In order for identification bias to occur in a randomized trial, the randomly assigned intervention must affect who is identified for inclusion in the statistical analysis of the study outcome [36]. For the effectiveness objective, an analysis that includes patients diagnosed with OUD after randomization meets this condition for the reasons described above. Thus, the effectiveness objective may be susceptible to identification bias if patients identified using post-randomization data are included in analyses (see Table 1).

To address the competing goals of fully capturing intervention effects with mitigating identification bias for the effectiveness objective, in the statistical design stage we considered several options for defining the study population to be used in the analysis. Hereafter, we will use the term “analytic sample” to refer to the population that would be included in an intent-to-treat statistical analysis for the effectiveness outcome. Our framework to address identification bias in the statistical study design then comprised the following steps.

- *Step 1. Identification of candidate analytic samples.* Consider different options for the choice of analytic sample to be used for the primary analysis. Determine which options include patients identified using post-randomization data (and are therefore potentially affected by identification bias) and which rely solely on pre-randomization data (and therefore avoid identification bias).
- *Step 2. Choice of unbiased analytic sample for the primary analysis.* Conduct a power analysis to identify which of the unbiased, pre-randomization samples identified in Step 1 achieves the maximal power. Use this sample for the primary analysis. Details on the methods used in the PROUD case study are below.
- *Step 3. Determine whether secondary analyses are needed to assess generalizability.* If generalizability of results from using the analytic sample identified in Step 2 is of concern (e.g. for the reasons described above), alternate samples that may be affected by identification bias may be considered as secondary analyses.
- *Step 4. Statistical analysis plan for secondary analyses in alternate analytic samples.* Specify a statistical analysis plan for secondary analyses that include individuals identified after randomization that accounts for the potential for identification bias.

#### Power evaluation to select an unbiased analytic sample for the primary effectiveness analysis

Here we describe the statistical methods used in our case study to select a primary analytic sample for the effectiveness objective that avoids identification bias by using pre-randomization data (Step 2). Because the goal of the power evaluation was to provide a general comparison of the operating characteristics of the different choices of analytic sample, we used a closed-form sample size formula based on Poisson regression [37] that did not account for the cluster-randomized nature of PROUD (Appendix of the Additional File). The effective sample size in a cluster-randomized study is equal to the actual sample size multiplied by a factor that depends on the within-cluster correlation [38]; thus, accounting for within-cluster correlation would reduce the power for all of the analytic samples under consideration, but we do not expect it to impact the relative performance of the power across these different samples (i.e. we assumed that clustering would affect each of the scenarios in the same way). We note, however, that once the primary analytic sample was selected based on the formula-based power evaluation described in this case study, the final power analysis for the PROUD Trial used a simulation approach that accounted for the within-cluster correlation (results not shown as not relevant to this case study and are described elsewhere) [30, 31].

For the formula-based power evaluation, we considered different scenarios that varied the true prevalence of OUD in the sites (parameterized by π) and we calculated power across a range of effect sizes for five different options for the analytic sample. Based on prior literature on the prevalence of OUD, which suggested that the true prevalence is approximately 1% in general US populations, with higher rates in certain subpopulations, study investigators hypothesized a likely range of values of 1%–4% to be used in the power evaluation (π = 0.01, 0.02, and 0.04). The five different options for the analytic sample corresponded to using the population of patients with a prior OUD diagnosis (Table 2, Sample 1), using the entire population of patients with a primary care visit (Table 2, Sample 2), and using a population of patients defined as being at “increased risk of OUD” based on known factors associated with OUD, such as taking prescribed high-dose opioids for chronic non-cancer pain (Table 2, Samples 3a, 3b, and 3c). Each of these scenarios were defined by particular values of sensitivity and specificity, which are shown in Table 3. In this context, sensitivity is the probability that an individual in the site is included in the analytic sample given that they have true OUD (documented or undocumented) and specificity is the probability that an individual in the site is not included in the analytic sample given that they do not have true OUD (i.e. 1 – specificity is the probability that they are included in the analytic sample even if they do not have OUD). Because the true sensitivity and specificity of different definitions of “increased risk” of OUD are not known in general primary care populations, we examined a range of different values informed by work in this area in one healthcare system [39], including a “high specificity” scenario (Table 2, Sample 3a), a “high sensitivity” scenario (Table 2, Sample 3b), and a scenario with equal sensitivity and specificity (Table 2, Sample 3c). Complete details of the formula-based power evaluation are in the Additional File.

**Table 2:** Considerations in using different sets of eligibility criteria for defining the analytic sample

Analytic sample	General considerations	Implications for effectiveness outcome No. days of acute care utilization (patient-level outcome)
Analytic samples not affected by identification bias^a^		
Sample 1. Patients with documented OUD before randomization	• Misses potential improvements in outcomes attributable to the intervention among new patients with OUD who were attracted to receive care after randomization due to the PROUD intervention. These patients could comprise a substantial proportion of patients with OUD treated due to the PROUD intervention (70%–90%) • Patients with a prior documented OUD may not reflect the general population of patients with true OUD^b^ • Given that OUD is underdiagnosed, restricting to patients with documented OUD before randomization reduces the sample size and therefore power for patient-level outcomes, relative to an open cohort design that includes those diagnosed after randomization (Sample 4 below)	Estimates of intervention effects within this select population of patients with documented OUD before randomization may not generalize to the broader population of individuals with true OUD who may be treated as part of the intervention (and therefore may not detect the true benefit).
Sample 2. All patients with primary care visits before randomization	• Same as bullet #1 for Sample 1 above • Sample includes patients with undocumented OUDs before randomization who might benefit from the intervention • Sample also includes many individuals without true OUD who would not be impacted by the intervention	• Since most individuals in the site population do not have OUD, the effect of the intervention on acute care utilization would be diluted, resulting in attenuation of the treatment effect toward the null • Relative to Sample 1, power could either be increased due to the higher sample size of patients with OUD or decreased due to including patients without true OUD in the analysis
Sample 3. Patients with primary care visits before randomization who have documented OUD or are at “increased risk” of OUD^c^	• Same as bullet #1 for Sample 1 above • Need to develop a definition of “increased risk” of OUD that seeks to include as many patients who truly have OUD (maximizing sensitivity) while limiting the number of patients included who do not truly have OUD (maximizing specificity). For example, this definition could be selected to target a high specificity (Sample 3a), a high sensitivity (Sample 3b), or a balanced sensitivity and specificity option (Sample 3c)	• Relative to Sample 1, results in a larger sample size of patients with true OUD (higher sensitivity). This could increase power • At the same time could lead to attenuated intervention effect estimates relative to Sample 1, since more of the identified individuals would not have OUD (lower specificity)
Analytic samples potentially affected by identification bias^a^		
Sample 4. Patients with documented OUD (before and/or after randomization)	• Patients diagnosed after randomization in the intervention arm may not be comparable to patients diagnosed after randomization in the control arm • Diagnosis of newly recognized OUD is expected to continue over time. Consequently, including individuals diagnosed after randomization could increase the sample size (and therefore power) as compared to Sample 1	Individuals diagnosed with OUD after randomization in the intervention arm are likely to be different (either sicker or healthier) with respect to their propensity for acute care utilization than individuals diagnosed with OUD after randomization in the control arm. This could lead to bias (see Table 1).
Sample 5. All patients with primary care visits (before and/or after randomization)	• Patients new to intervention sites after randomization may not be comparable to patients new to the control sites after randomization • As in Sample 2, sample includes many individuals without true OUD who would not be impacted by the intervention • Captures outcomes of all patients with OUD who could be treated: patients seen previously in the clinic (including those with and without documented OUD before randomization) and those attracted to receive care as part of the PROUD intervention	As in Sample 2, the effect of the intervention would be diluted in the entire site population relative to Samples 1 or 4 and power could either be increased or decreased.
Sample 6. Patients with primary care visits who have documented OUD or are at “increased risk” of OUD^c^ (before and/or after randomization)	• Patients identified as at “increased risk” of OUD after randomization in the intervention arm may not be comparable to patients identified as at “increased risk” of OUD after randomization in the control arm • As in Sample 3, need to develop a definition of “increased risk” of OUD that seeks to include as many patients who truly have OUD while limiting the proportion of patients who do not truly have OUD who are included	As in Sample 3, results in a larger sample size of patients with true OUD, but also could include many patients without OUD; thus, power could either be increased or decreased relative to Sample 4.

*EHR* electronic health record, *OUD* opioid use disorder

^a^ Identification bias is a form of selection bias that can occur in open-cohort cluster-randomized trials when the randomized intervention group assignment affects who is identified as eligible for a particular analysis. Identifying eligibility for inclusion in trial analyses before randomization (or using data collected pre-randomization) avoids this source of bias

^b^ Documented OUD refers to patients with an OUD diagnosis documented in the EHR; True OUD refers to patients with OUD regardless of its recognition by clinicians and/or documentation in the EHR

^c^ Planned definition of “increased risk” of OUD included individuals with any documented OUD diagnosis at baseline or anyone with both chronic opioid therapy (outside of end of life, palliative care, or active cancer treatment) and at least one of the following risk factors associated with increased risk of OUD: high morphine equivalent dose, alcohol or other substance use disorders, mental health disorders, concurrent sedative use, or pain in two or more body regions (e.g. headache and back pain)

**Table 3:** Assumed values of sensitivity and specificity for each analytic sample using pre-randomization data considered in the power evaluation

				True OUD prevalence (π)		
				0.01	0.02	0.04
Sample^a^		Assumptions / Notes	Specificity	Sensitivity^b^		
1	Documented OUD	• Assumes all individuals with documented OUD do in fact have true OUD (specificity = 1) • Sensitivity selected to be consistent with the observed proportion of patients with documented OUD based on Phase 1 data (0.5%) and the specific choice of the true prevalence (π)	1	0.5	0.25	0.125
2	All patients	By definition, sensitivity = 1 and specificity = 0	0	1	1	1
3a	High specificity	Selected to have slightly higher sensitivity than Sample 1 (1.2 times the value), at the cost of slightly reduced specificity	0.95	0.6	0.3	0.15
3b	High sensitivity	• Sensitivity was selected based on a previously developed algorithm^c^ to identify individuals with opioid abuse and addiction, among patients on long-term opioid therapy • We considered a lower specificity (0.5 vs 0.64^c^) given that our initial sample is the entire site population, not restricted to long-term opioid users	0.5	0.85	0.85	0.85
3c	Equal sens./spec.	Selected to have lower sensitivity and higher specificity than Sample 3b	0.6	0.6	0.6	0.6

*OUD* opioid use disorder

^a^ All options identify the study population using baseline (pre-randomization) data; see Table 2

^b^ For some of the options, sensitivity was allowed to vary across the assumed prevalence of true OUD (π)

^c^ Different cut-points for the developed risk score [39] achieved different values of sensitivity and specificity. We considered the “high sensitivity” scenario that achieved a sensitivity of 0.85 and a specificity of 0.64 in the validation sample (D. Carrell, personal communication, 5 July 2017)

## Results

### Step 1. Identification of candidate analytic samples for the primary effectiveness analysis

The set of different choices considered for the analytic sample are outlined in Table 2. Options differed based on whether the sample would be restricted to patients who could be identified before randomization versus allowing patients identified after randomization to enter the sample as well, and whether the sample would be restricted to patients with documented OUD or include a broader sample of patients (e.g. using the entire site population or patients at “increased risk” of OUD). Broadly, considerations guiding the choice of the analytic sample definition included the desire to capture the full effect of the intervention, to obtain unbiased estimates of the intervention effects, and to maximize study power to detect an effect.

Concern about the potential for identification bias (see Table 1), which could lead to bias in either direction (toward or away from the null), led study investigators to favor an analysis that avoided identification bias for the effectiveness objective by defining the analytic sample for the primary analysis using only pre-randomization data (Table 2, Samples 1–3). However, investigators were also concerned that restricting the analysis to individuals with documented OUD pre-randomization (Sample 1) would yield a relatively low sample size, whereas using the entire site population (Sample 2) in the statistical analysis could dilute the effect of the intervention because most people do not have OUD. This led the study team to consider a “middle ground” option in which baseline data would be used to identify individuals at “increased risk” of OUD (Sample 3). Relative to using the population of patients with documented OUD (Sample 1), such a middle ground option would include a larger number of patients with true OUD (i.e. higher sensitivity) at the cost of also including patients without true OUD in the sample (i.e. lower specificity).

### Step 2. Choice of unbiased analytic sample for the primary effectiveness analysis

To explore whether this middle ground option (Table 2, Sample 3) for an analytic approach not impacted by identification bias but with a larger sample size was likely to result in improved statistical power, we conducted an evaluation to compare the power under different analytic samples that could be identified using baseline (pre-randomization) data, as described above in the “Methods” section (see Table 3 for the sensitivity and specificity for each sample).

**Fig. 2:**
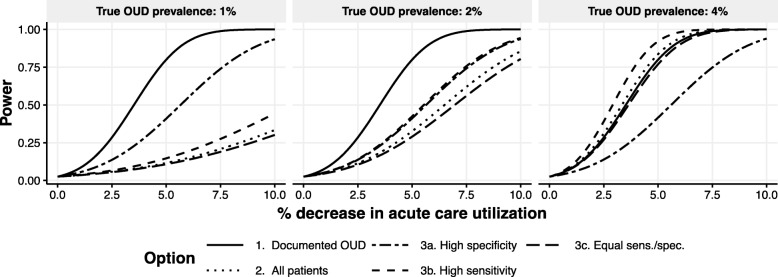
Comparison of statistical power across different options for the analytic sample for the effectiveness analysis. The x-axis shows the intervention effect size, parameterized as the percentage decrease in the expected number of days of acute care utilization comparing patients with OUD (recognized or unrecognized) in the intervention versus usual care arm. All options for the analytic sample (described in Table 2) use pre-randomization data. Each panel represents a different true prevalence of OUD (1%, 2%, or 4%). Options 3a, 3b, and 3c correspond to different assumptions of the properties of an algorithm for defining “increased risk” of OUD (see Table 3). Higher sensitivity includes more patients with true OUD whereas higher specificity excludes more patients without true OUD. Power calculations were based on closed form sample size formula based on Poisson regression (details are in the Additional File)

Figure 2 shows the results of the formula-based power evaluation for the five different samples considered (Samples 1, 2, and 3a, 3b and 3c), varying the prevalence of true OUD and the magnitude of the expected effect of OUD treatment on acute care utilization. Across the five options for the analytic sample, we found that Sample 1 (patients with documented OUD) maximized power if the prevalence was 1%–2%, whereas Sample 3b maximized power if the prevalence was 4%. This suggests that the optimal choice of analytic sample that balances the tradeoff of increasing sensitivity (which increases power) at the cost of decreased specificity (which decreases power) depends on the true prevalence of OUD. If study investigators had had strong prior knowledge of which of the values considered was most likely to be the true OUD prevalence in the PROUD sites (π), then the optimal analytic sample would be the one that maximized statistical power under that value of π. However, without strong prior knowledge on the true prevalence in the PROUD sites within this range of possible values, the study team decided to select Sample 1. Although other options for the analytic sample had higher power at the highest prevalence of true OUD considered (4%), this increase in power was not as large as the increase in power of Sample 1 relative to the other options when the prevalence was 1%–2%. Thus, Sample 1 appeared to be a good choice of unbiased sample for primary analyses across plausible values of the unknown true prevalence of OUD within the PROUD site populations.

### Step 3. Consider whether secondary analyses are needed to assess generalizability

Though the primary analysis of the effectiveness objective avoids the potential for identification bias, it may lack generalizability (Table 2). Specifically, individuals with an OUD diagnosis documented in their EHR before randomization may not reflect the general population of primary care patients with true OUD across the health systems. Additionally, the magnitude of benefit of the intervention might be underestimated in an intent-to-treat analysis within this sample if only a small proportion of the patients treated by the nurse had OUD documented before randomization. To address these limitations in the primary analytic sample, study investigators considered a secondary analysis using an open cohort that would allow individuals diagnosed after randomization to also be included (Table 2, Sample 4). However, since patients newly diagnosed with OUD in the intervention group after randomization may differ markedly from those newly diagnosed in the control group, statistical analyses within this secondary, more generalizable analytic sample must apply observational data methods to account for the potential for identification bias.

### Step 4. Statistical analysis plan for secondary analyses in alternate analytic samples

A range of potential statistical methods could be applied to adjust for identification bias, including regression adjustment, propensity score analyses, and instrumental variable methods. For our statistical analysis plan (detailed in the Protocol [30, 31]), we proposed adjusting the regression model for measured factors (e.g. demographic characteristics, co-morbidity) that are found to differ across patients who are newly diagnosed with OUDs after randomization in the intervention versus control groups. We also plan to investigate the potential for unmeasured factors to cause bias by including an interaction term in the model between the intervention group and the time period at which the person was first diagnosed with OUD. This will allow us to estimate separate intervention effects among individuals who were identified for inclusion in the analytic sample based on pre-randomization data, and individuals who were identified for inclusion in the analytic sample based on post-randomization data (including some who had been in the site without documented OUD before randomization and others with OUD new to the site after randomization) after adjusting for measured confounders (e.g. pre-randomization value of the outcome). A clinically meaningful difference in the estimated effects across these groups of patients stratified by the timing of their first OUD diagnosis (before or after randomization) could either reflect a true difference in the intervention effect, or, more likely, it could reflect the impact of unmeasured factors that differ between patients newly diagnosed after randomization in the intervention arm versus those newly diagnosed after randomization in the control arm.

## Discussion

The present case study highlights the potential impact of identification bias in pragmatic, cluster-randomized trials with open-cohort designs and how this source of bias is being addressed in a study using EHR data to evaluate the impact of a primary care OUD treatment program on reducing acute care utilization among patients with OUD. We addressed identification bias in the statistical design by selecting a primary analytic sample that used only pre-randomization data and we proposed secondary analyses that would allow individuals identified with OUD after randomization to be included, thereby capturing more of the potential effect of the intervention. Because such secondary analyses may be affected by identification bias, the proposed analytic approach uses regression-based methods to adjust for measured factors that could differ between patients newly identified in the intervention and control arms, along with a sensitivity analysis to investigate the potential impact of unmeasured confounders.

In determining the analytic sample for the effectiveness aim using pre-randomization data, it was initially thought that identifying individuals at “increased risk” of OUD would have higher power relative to using the entire population of primary care patients or restricting to those patients with a prior diagnosis. However, the results of our evaluation suggested that this was not always the case. In particular, using the population of individuals with documented OUD at baseline had higher power under scenarios of low (1%) or moderate (2%) prevalence of true OUD compared to the other options considered. This finding demonstrates the importance of conducting preliminary analyses to evaluate different choices for the analytic sample. Prior information on the properties of algorithms used to identify the analytic sample (e.g. sensitivity, specificity [39]), along with knowledge of the true prevalence of the latent disease (here OUD), can inform such evaluations.

While the framework considered in this paper prioritized selecting a population using pre-randomization data for the primary analysis, other studies have taken alternate approaches [17]. One example is a pragmatic, open-cohort, stepped-wedge trial to evaluate a program integrating alcohol-related care into primary care [40]. A main objective of that prior trial was to test whether the intervention increased treatment for alcohol use disorders documented in the EHR as compared to usual primary care. Identification bias was a major concern, because the intervention was expected to alter the number and characteristics of patients being diagnosed with alcohol use disorders. Avoiding identification bias by restricting the analysis to patients diagnosed before randomization was not appealing because of the nature of the stepped wedge design, whereby patients seen in the clinic before randomization may not have follow-up visits to the clinic during the time period after the clinic crossed over to the intervention group (up to 2–3 years later). Consequently, investigators favored a primary analysis approach that used as its study population all patients who visited the clinic (including patients with a first visit after randomization), because the intervention was not expected to affect this population [40, 46, 47]. Another example is a cluster-randomized, parallel group trial to test the effectiveness of an intervention to improve colorectal cancer screening rates [41, 42]. Because the primary analytic sample included all patients who were not up to date with colorectal cancer screening guidelines (including patients who became out of date during the post-randomization period), the analysis had the potential to be affected by identification bias [17]. The study addressed this issue analytically by considering regression-based adjustment for measures hypothesized to be associated with the outcome, along with a sensitivity analysis within an alternate study sample not expected to be affected by identification bias [17]. The particular study objective of the trial may also play a role in the choice of approach to address identification bias. Although not the focus of the current case study, the implementation objective of PROUD to evaluate whether the intervention increased provision of OUD medication treatment by necessity required using an open cohort that included patients entering after randomization, because a key element of the intervention is that it attracts new patients into primary care who are seeking treatment for OUD. In general, the choice of which analytic sample to use for the primary analysis is likely to depend on a range of factors, including the study objective, the study design (e.g. parallel group vs stepped wedge), type of outcome, and assumptions on the mechanism by which patients could be differentially identified across intervention groups.

This work has several limitations. First, because the PROUD Trial is still in the data collection phase, we are not yet able to evaluate how the choice of analytic sample would affect final inferences in the effectiveness analysis. This case study was therefore intended to highlight issues relating to identification bias for others designing pragmatic trials. Second, although the framework proposed here illustrates how to address identification bias within a given case study, there remain several gaps in knowledge on the optimal statistical design to address identification bias in open-cohort pragmatic trials more generally. Future work could evaluate the operating characteristics of these choices by conducting comprehensive simulation studies across a wide range of scenarios. Third, our proposed secondary analysis that includes patients newly identified with OUD after randomization uses just one of many possible analytic approaches to account for identification bias, regression adjustment. Principal stratification is an alternate approach that could be adopted in this setting [43]. For example, one could consider a pre-randomization subgroup (principal stratum) that consists of patients who would be diagnosed with OUD after randomization if assigned to the intervention arm but who would not be diagnosed with OUD if assigned to the control arm, and estimate the effect of the PROUD intervention among this subgroup using instrumental variable methods [44]. Such an approach appears promising but has not yet been fully developed for the cluster-randomized trial setting. Future work is needed to develop and compare alternate analytic strategies to address post-treatment selection bias when including patients identified after randomization.

## Conclusion

In the present paper, we proposed a framework for addressing identification bias in pragmatic cluster-randomized trials. In the study design, identification bias can be avoided by specifying the analytic sample using baseline (pre-randomization) data. In the analysis plan, methods can be applied to adjust for identification bias and alternate analytic samples that include participants who enter the study after randomization can be considered in sensitivity analyses. With more studies seeking to leverage existing data sources such as EHRs to make clinical trials more affordable and generalizable, the potential for identification bias is likely to become increasingly common in the future. The framework developed in this case study can serve as a model for any pragmatic trial seeking to mitigate this source of selection bias due to the intervention affecting identification of the population included in trial analyses.

## Supplementary information

**Additional file 1.** Details of the power evaluation for selecting an unbiased analytic sample for the PROUD effectiveness analysis.

## Data Availability

The datasets used in the current study are available from the corresponding author on reasonable request.

## Abbreviations

EHR: Electronic health record
OUD: Opioid use disorder
PROUD: Primary care Opioid Use Disorders treatment (PROUD) trial
