# Supplementary material for: Addressing identification bias in the design and analysis of cluster-randomized pragmatic trials: a case study
Source: Trials. 2020 Mar 23;21:289. doi: 10.1186/s13063-020-4148-z (PMC7092580; doi:10.1186/s13063-020-4148-z)
Supplement: Supplementary file 1 — Additional file 1. Details of the power evaluation for selecting an unbiased analytic sample for the PROUD effectiveness analysis. [file 13063_2020_4148_MOESM1_ESM.docx]

Additional File 1 for the article “Addressing identification bias in the design and analysis of cluster-randomized pragmatic trials: a case study”

**Appendix.** Details of the power evaluation for selecting an unbiased analytic sample for the PROUD effectiveness analysis

Here we provide the details on how power was calculated as part of the evaluation to compare different choices for defining the analytic sample (using pre-randomization data) for the effectiveness outcome (Figure **2** of the main text). The analytic sample refers to the study population included in the analysis and is identified from the base population of patients who visit primary care clinics pre-randomization. For the purpose of this comparison, we considered the simpler scenario of an individually randomized trial with two treatment groups (control group and intervention group). We define the following parameters:

| **Parameter** | **Definition** | **Assumed value(s)** | **Explanation** |
| --- | --- | --- | --- |
| π | Prevalence of true OUD in the population | 0.01, 0.02, 0.04 |  |
| $N$ | Total sample size of all patients who visit the participating primary care sites (across both intervention groups) | 300,000 | Approximate number of individuals with a visit to the 12 PROUD sites during a 3-year baseline period pre-randomization |
| $\mu_{c}$ | Average number of acute care days (over a 2-year follow-up period after randomization) among patients with true OUD in the control sites | 8 | Estimated based on Phase 1 data from individuals with a documented OUD diagnosis among the 12 PROUD sites |
| $\mu_{0}$ | Average number of acute care days (over a 2-year follow-up period after randomization) among patients without true OUD (assumed to be the same in both control and intervention sites) | 2 | Estimated based on Phase 1 data from individuals without a documented OUD diagnosis from the 12 PROUD sites |
| $RR_{OUD}$ | Treatment effect among patients with true OUD (not necessarily previously diagnosed), defined as the relative risk comparing the expected number of acute care days among OUD patients in the intervention arm to the expected number of acute care days among OUD patients in the control arm | From 0.8 to 1 | - A range of effect sizes was considered for the treatment effect. Note that this parameter corresponds to an intent-to-treat effect, since not all patients with OUD may actually receive treatment from the nurse - For the purpose of presenting results (as shown in Figure 2), we parameterize the effect size as $100\%\left( 1- RR_{OUD} \right)$, which corresponds to the percent decrease in acute care utilization among patients with OUD |
| $s$ | Sensitivity | See **Table 3** in main text | Probability that an individual is included in analytic sample given that they have true OUD |
| $\sigma$ | Specificity | See **Table 3** in main text | Probability that an individual is not included in the analytic sample given that they do not have true OUD |
| $z_{\alpha/2}$ | Critical value (assumed to be $>0$) from standard normal distribution corresponding to a level $\alpha$ two-sided test | $\alpha=0.05$ | Conventional level |

Based on the above parameters, we derived additional parameters that summarize relevant statistics on the identified analytic sample:

| **Derived parameter** | **Formula** | **Explanation** |
| --- | --- | --- |
| $PPV$ | $\frac{\pi s}{\pi s+\left( 1-\pi\right)(1-\sigma)}$ | Positive predictive value: probability that an individual has true OUD given that they are included in the analytic sample |
| $n$ | $\frac{N\pi s}{PPV}$ | Total sample size of analytic sample (across both intervention groups) |
| $\tilde{\mu_{c}}$ | $PPV\mu_{c}+\left( 1-PPV \right)\mu_{0}$ | Average number of acute care days (over a 2-year follow-up period) among patients in the analytic sample in the control group |
| $\tilde{RR}$ | $\frac{PPV\mu_{c}RR_{OUD}+\left( 1-PPV \right)\mu_{0}}{PPV\mu_{c}+\left( 1-PPV \right)\mu_{0}}$ | Treatment effect among patients in the analytic sample, defined as the relative risk comparing the expected number of acute care days among identified patients in the intervention arm to the expected number of acute care days among identified patients in the control arm |

Then, to compute the power (as shown in **Figure 2** of the main text), these derived parameters were used within the power formula for Poisson regression ^36^ for the scenario of equal sample sizes in the two treatment groups,

$$Power=\Phi\left( \frac{\sqrt{n\tilde{\mu_{c}}} \left| \log\tilde{RR} \right|-2z_{\alpha/2}}{\sqrt{2+2/\tilde{RR}}} \right)$$
